# The unresolved safety concerns of bovine thrombin

**DOI:** 10.1186/1754-9493-2-23

**Published:** 2008-09-22

**Authors:** Aryeh Shander, Mazyar Javidroozi

**Affiliations:** 1 Anesthesiology, Critical Care and Hyperbaric Medicine, Englewood Hospital and Medical Center, Englewood, USA

## Abstract

A recent review has suggested that bovine thrombin is not associated with an increased risk of bleeding in surgical populations. In spite of extremely limited evidence available, many valuable resources (e.g. safety surveillance and post-marketing programs, case reports) were excluded in reaching this conclusion. While waiting for the adequately powered, controlled clinical trials to address the effects of bovine thrombin on bleeding and thrombotic events, the potential risk cannot be simply ignored. Rather, continued vigilance in the post-surgical setting for bleeding events that may be associated with the development of acquired coagulation factor inhibitors following bovine thrombin administration is warranted.

## 

A recent review by Reynolds and colleagues examined the rate of bleeding events in surgical patients using data from 16 clinical studies published and indexed in Medline from 1997 to 2007 in which risk of bleeding was a primary or secondary objective [1]. Five additional surgical studies that evaluated topical thrombin were also included, although risk of bleeding was not required to be a primary or secondary endpoint. Based on the defined literature review, the authors concluded that the risk of bleeding was not different between patients treated with bovine thrombin compared with patients in other surgical populations.

Intractable surgical bleeding is a rare event and its association with poor outcome suggests significant under-reporting. Although rare, this complication has an enormous clinical and financial burden [2]. Based on the current published data, it is not possible to draw any definitive conclusions on the safety of bovine thrombin. Randomized controlled studies to establish safety are needed but the required number of subjects is related to the frequency of the event in question. As such, a huge number of subjects need to be recruited making this study almost unattainable.

The available studies cited in this review are indicators of severely llimited evidence on the safety of topical bovine thrombin. It is hard to compare the bleeding rates among independent studies on different patient populations undergoing various procedures as several factors can play a role. In the absence of (and waiting for) evidence of higher quality, we need to draw on any and all available evidence.

Safety surveillance and/or pharmacovigilance post-marketing programs are a condition of product approval. Invariably, the safety profile of a marketed product continuously evolves to reflect accumulated clinical experience. Despite reduced reliability compared with evidence from trials, such programs, in addition to published case reports, are important tools for further identifying and assessing potential safety concerns. In the case of topical bovine thrombins and despite very limited available data, Reynolds et al excluded these resources in their assessment, resulting in a skewed and deficient evaluation of the association between use of topical bovine thrombin and bleeding events. In fact, a boxed warning has been included in the prescribing information for topical bovine thrombin based on data from clinical trials, numerous early sentinel case reports, and post-marketing adverse event surveillance [3]. This boxed warning describes the occasional association of abnormalities in hemostasis, which appear to be related to the formation of bovine thrombin product antibodies and subsequent cross-reaction with endogenous proteins. These antibodies have been largely linked to impurities present in bovine thrombin preparations (e.g. prothrombin, factors V, VII and X) and recent reports indicate that improved manufacturing process can significantly reduce these and other impurities [4]. Nonetheless, these processes cannot eliminate all impurities [5], and more importantly, even a hypothetical completely pure bovine thrombin preparation will still pose some immunogenicity because of species-specific epitopes, resulting in production of anti-bovine antibodies with some degree of of cross-reacting with human thrombin [6,7].

Although a pharmacoeconomic assessment has not been conducted, a cursory examination of three recent case reports suggests that bovine thrombin-associated bleeding events may have a substantial impact on patient outcomes and institutional costs [8-10]. Interventions reported in these cases included corticosteroids and intravenous immunoglobulin (IVIG) as well as fresh frozen plasma, platelets and red blood cell transfusion, plasmapheresis, vitamin K, recombinant factor VIIa (rFVIIa), cyclophosphamide, and/or rituximab; thus, the potential economic impact of an induced coagulopathy associated with exposure to bovine thrombin may be significant.

Based on the incomplete inclusion of available data and limitations of the selected clinical trials in their review, we believe the conclusion determined by Reynolds and colleagues is unsubstantiated. Furthermore, in the absence of large, controlled clinical trials specifically designed and adequately powered to detect the effect of bovine thrombin on bleeding and thrombotic risk, the potential risk cannot be simply ignored. In fact, the presence of risk associated with bovine thrombin application is not controversial; the question to resolve is what the incidence of these events in routine surgical practice is. We believe that for now, the full body of evidence, including clinical trial results, case studies, rigorous and comprehensive review articles, and spontaneous adverse event reports, taken collectively supports continued vigilance in the post-surgical setting for bleeding events that may be associated with the development of acquired coagulation factor inhibitors. We believe that the full body of evidence-including clinical trials results, case studies, rigorous and comprehensive review articles, and spontaneous adverse event reports-taken collectively, the potential immediate and long-term costs associated with treating bovine thrombin-induced coagulopathies make it reasonable and in line with patients' interests to consider other commercially available and less immunogenic forms of thrombin as prudent alternatives.

## Abbreviations

IVIG: intravenous immunoglobulin; rFVIIa: recombinant factor VIIa.

## Competing interests

Dr Shander has received honoraria, consulting fees and research grants from Biopure, Alliance Pharmaceuticals, Hemosol BioPharma, Abbott Laboratories Hospira and ZymoGenetics. Dr Javidroozi reported no competing interest.

## Authors' contributions

AS conceived and developed the initial draft. MJ reviewed the literature and developed the final draft. Both read and approved the final letter.
